# Supplementary figures and images for: The three-dimensional flower-like Bi2WO6 assisted by ethanolamine through a microwave method for efficient photocatalytic activity
Source: R Soc Open Sci. 2019 Mar 20;6(3):181422. doi: 10.1098/rsos.181422 (PMC6458385; doi:10.1098/rsos.181422)

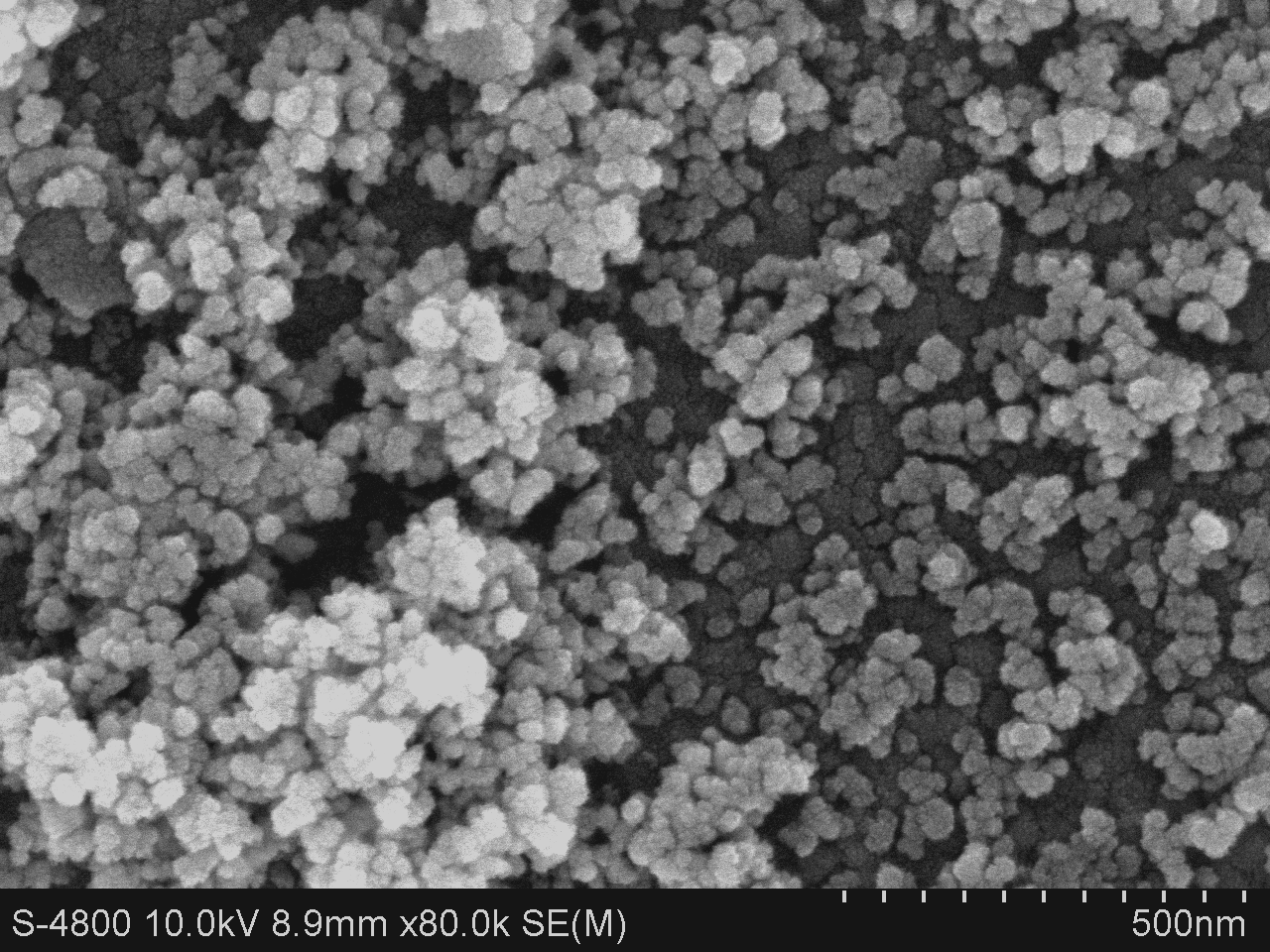

Supplement: 1.tif [file rsos181422supp6.tif]

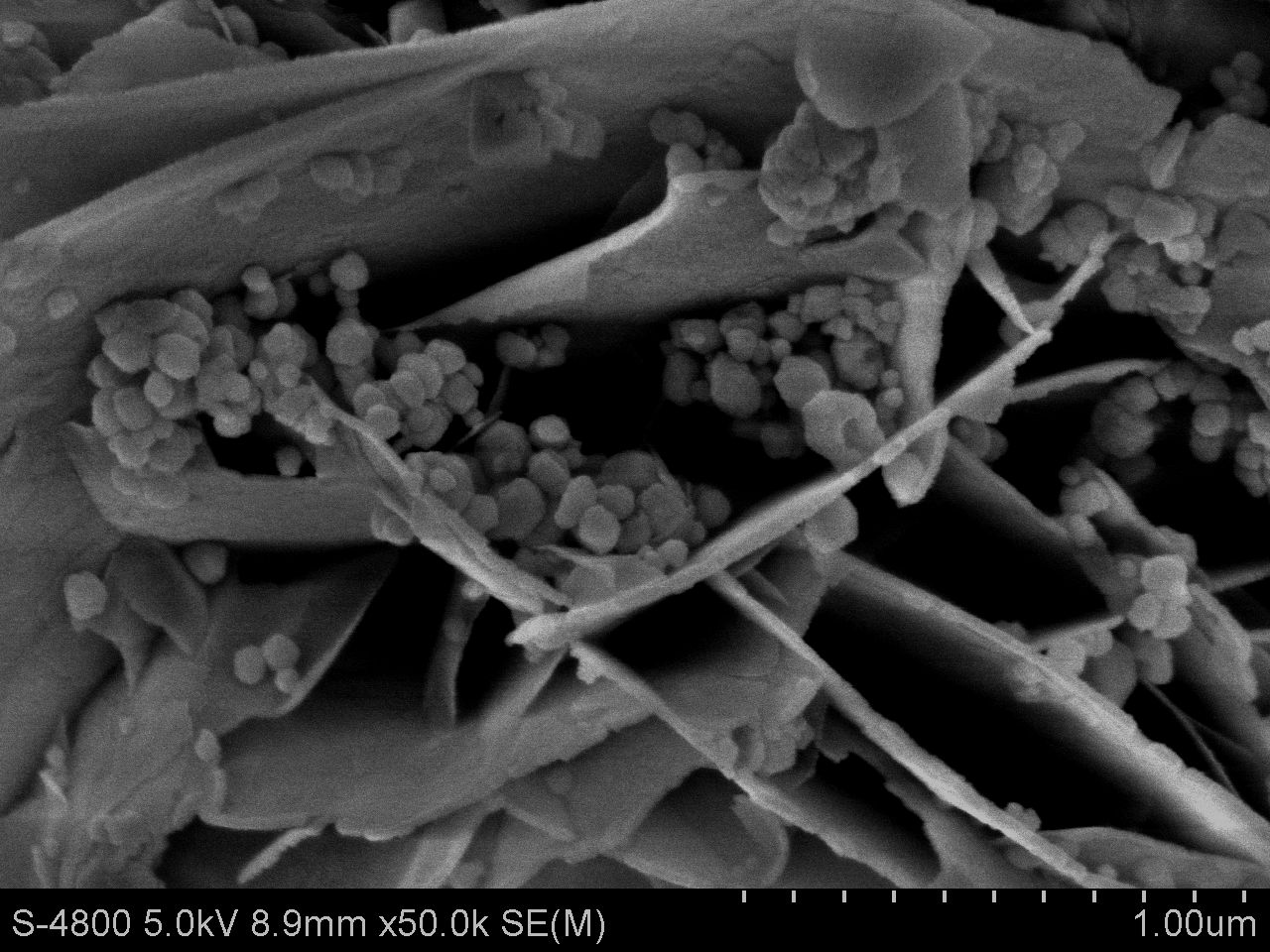

Supplement: 2.tif [file rsos181422supp8.tif]

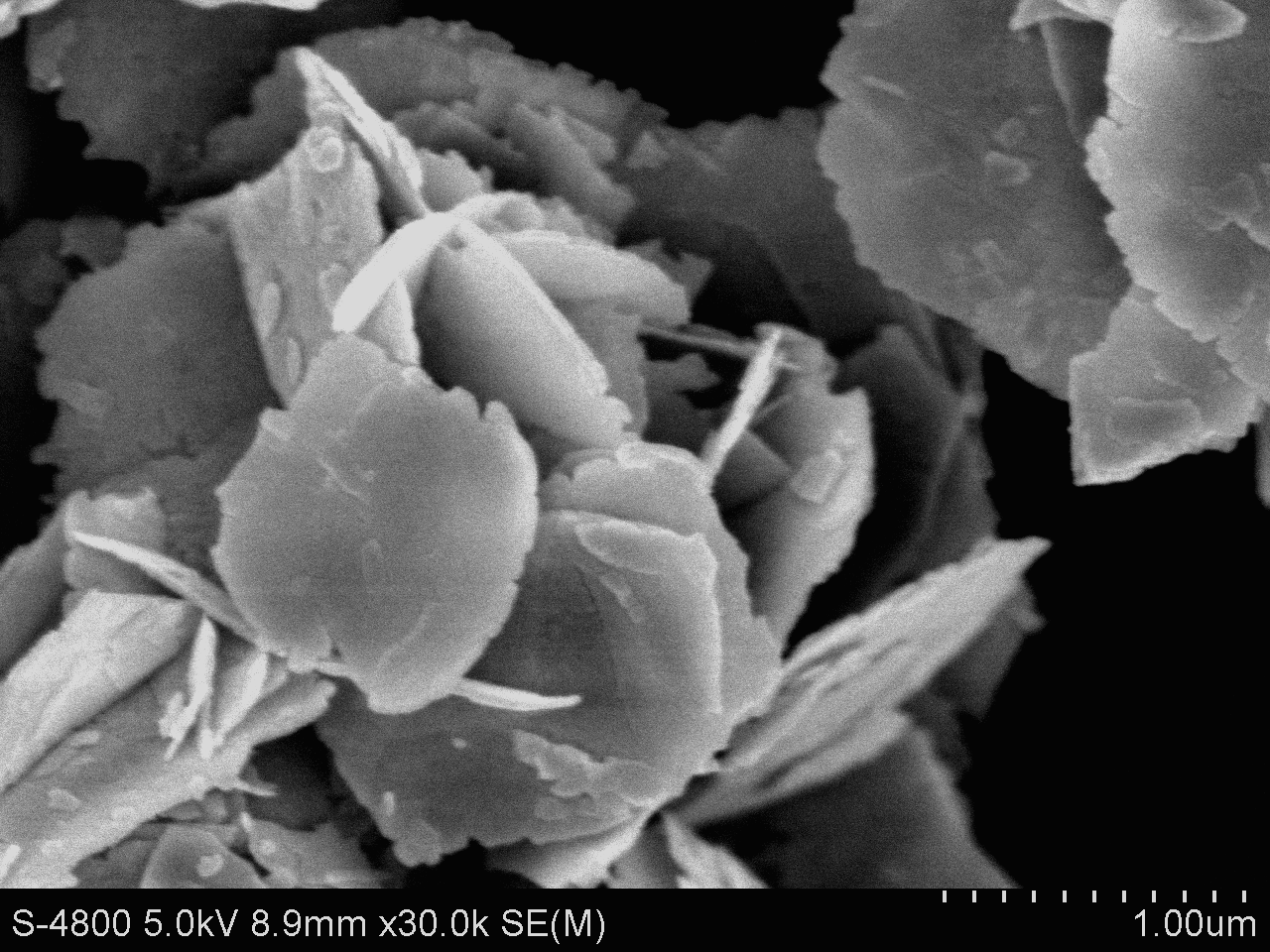

Supplement: 3.tif [file rsos181422supp9.tif]

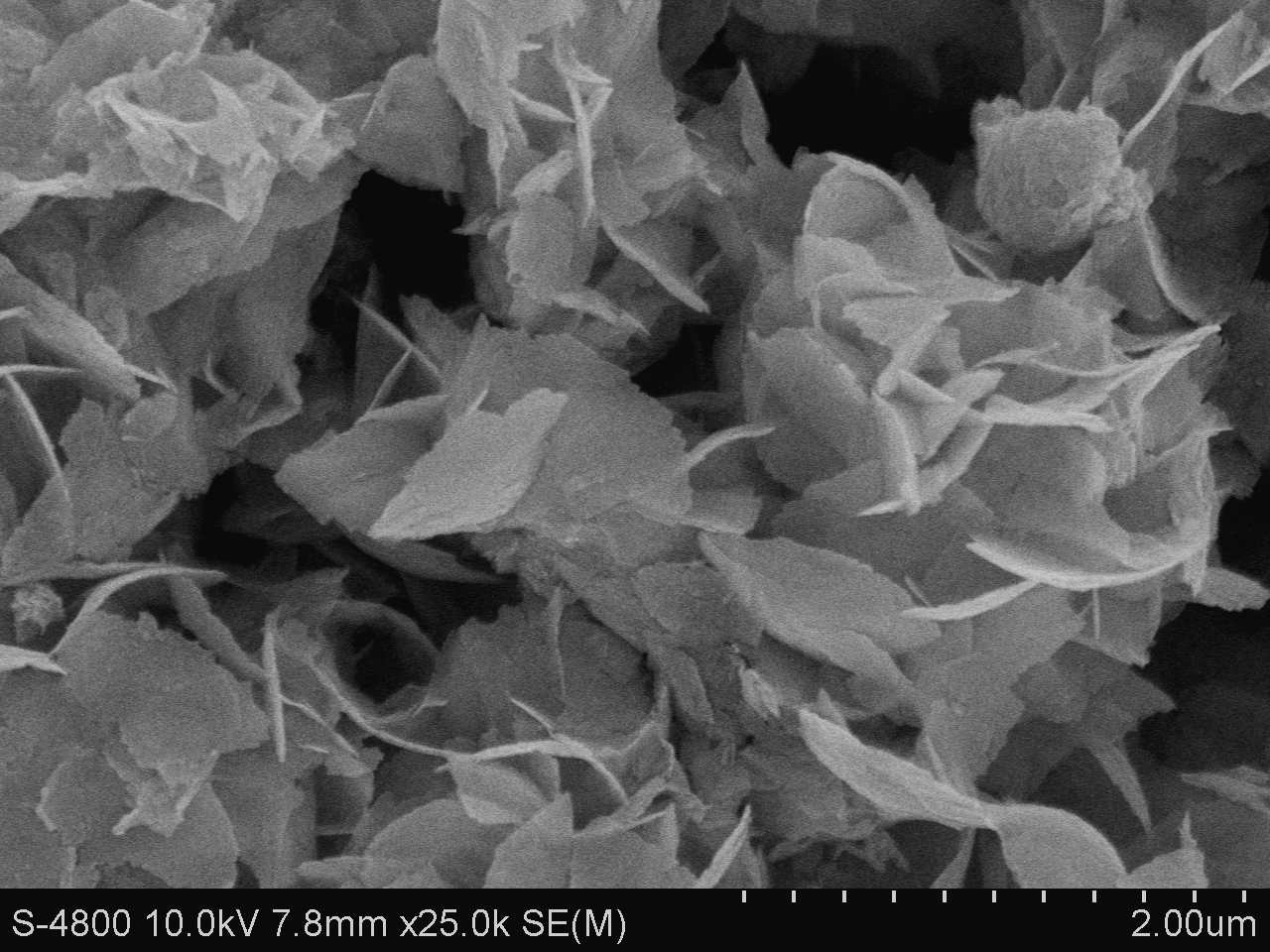

Supplement: 4.tif [file rsos181422supp11.tif]

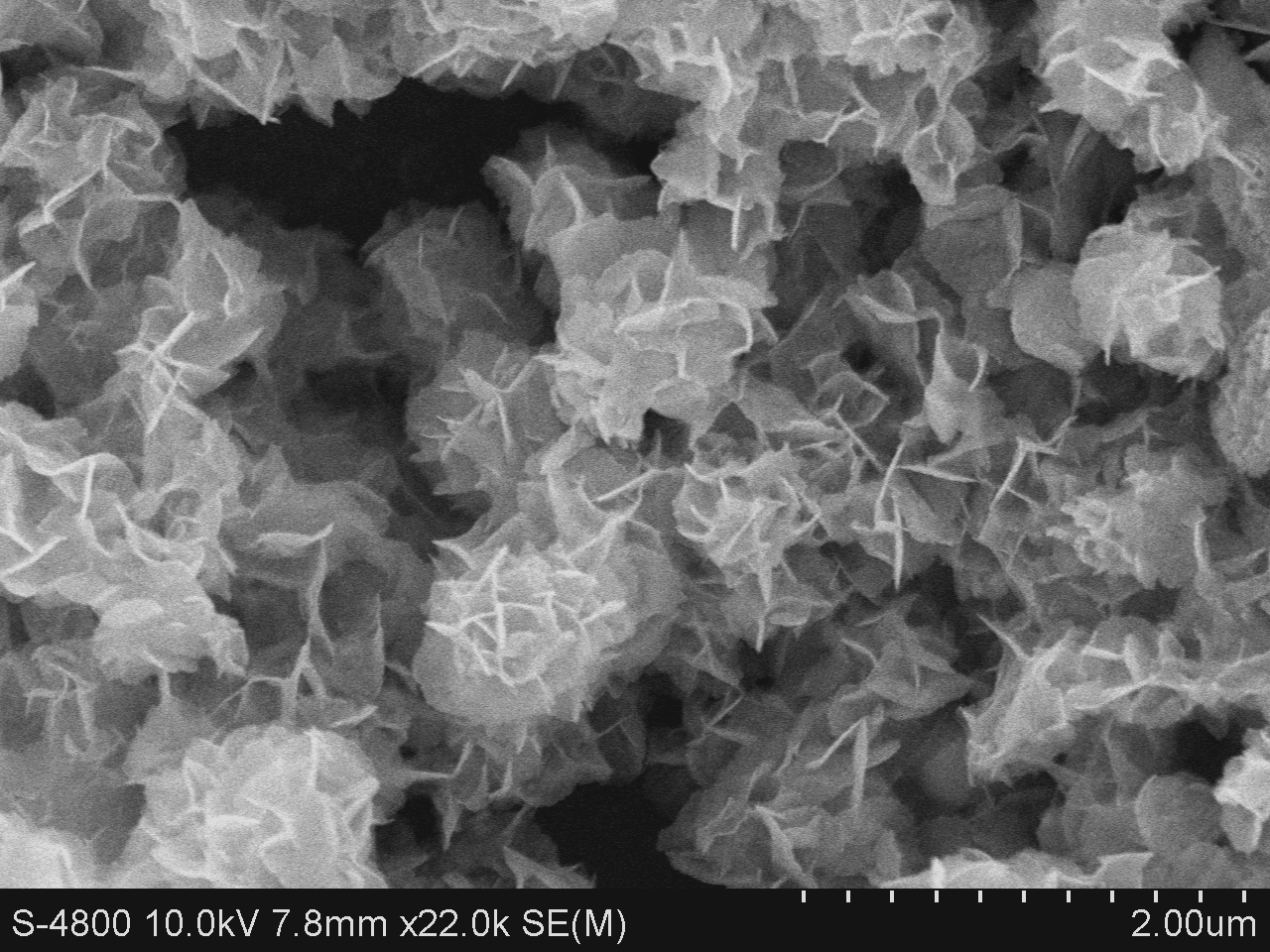

Supplement: 5.tif [file rsos181422supp13.tif]
